# Barrier crossings and winds shape daily travel schedules and speeds of a flight generalist

**DOI:** 10.1038/s41598-021-91378-x

**Published:** 2021-06-08

**Authors:** Lina Lopez-Ricaurte, Wouter M. G. Vansteelant, Jesús Hernández-Pliego, Daniel García-Silveira, Ana Bermejo-Bermejo, Susana Casado, Jacopo G. Cecere, Javier de la Puente, Fernando Garcés-Toledano, Juan Martínez-Dalmau, Alfredo Ortega, Beatriz Rodríguez-Moreno, Diego Rubolini, Maurizio Sarà, Javier Bustamante

**Affiliations:** 1 Departament of Wetland Ecology, Estación Biológica de Doñana (EBD-CSIC), C/Américo Vespucio 26, E-41092 Seville, Spain; 2 Freelance, Independent Researcher, Seville, Spain; 3 Bird Monitoring Unit, SEO/BirdLife, C/Melquiades Biencinto 34, 28053 Madrid, Spain; 4 Terra Naturalis, C/Uno 41, 28514 Madrid, Spain; 5 Area Avifauna Migratrice, Istituto Superiore Per la Protezione e la Ricerca Ambientale (ISPRA), Via Ca′ Fornacetta 9, 40064 Ozzano dell′Emilia BO, Italy; 6 GREFA, Monte del Pilar S/N, 28220 Majadahonda, Madrid, Spain; 7 Dipartimento di Scienze e Politiche Ambientali, Università Degli Studi di Milano, Via Celoria 26, 20133 Milan, Italy; 8 Dipartimento STEBICEF, Università Degli Studi di Palermo, Via Archirafi 18, 90123 Palermo, Italy

**Keywords:** Animal migration, Behavioural ecology

## Abstract

External factors such as geography and weather strongly affect bird migration influencing daily travel schedules and flight speeds. For strictly thermal-soaring migrants, weather explains most seasonal and regional differences in speed. Flight generalists, which alternate between soaring and flapping flight, are expected to be less dependent on weather, and daily travel schedules are likely to be strongly influenced by geography and internal factors such as sex. We GPS-tracked the migration of 70 lesser kestrels (*Falco naumanni*) to estimate the relative importance of external factors (wind, geography), internal factors (sex) and season, and the extent to which they explain variation in travel speed, distance, and duration. Our results show that geography and tailwind are important factors in explaining variation in daily travel schedules and speeds. We found that wind explained most of the seasonal differences in travel speed. In both seasons, lesser kestrels sprinted across ecological barriers and frequently migrated during the day and night. Conversely, they travelled at a slower pace and mainly during the day over non-barriers. Our results highlighted that external factors far outweighed internal factors and season in explaining variation in migratory behaviour of a flight generalist, despite its ability to switch between flight modes.

## Introduction

The ability to fly gives birds the unique capacity to perform fast seasonal movements up to thousands of kilometres a year across multiple and often inhospitable habitats^1^. Migrants often show great spatio-temporal flexibility in migratory behaviour throughout these challenging journeys^2^. That flexibility is governed by an interplay between (1) external factors such as weather conditions and geography that influences connectivity and creates so-called ecological barriers^3–5^; and (2) internal factors such as motion capacity (dependent on, for example, wing morphology), orientation ability, and the individual’s age, sex, and/or reproductive state that shape the internal motivation to move^3^. However, understanding the relative contributions of such external and internal factors influencing migratory behaviour is often hampered by the lack of high-resolution tracking data for a diverse sample of individuals^6,7^.

Studies that take into account the interplay of external and internal factors in shaping migratory behaviour (commonly measured via metrics such as ground speed, travel distance, duration of stopovers, and route straightness^8–10^) present a bias towards large soaring birds. Studies on these species have demonstrated that variation in weather (e.g., winds, thermals, and orographic updrafts) is often the prevailing factor explaining migration patterns, such as seasonal and regional differences in hourly and daily speeds^8,11^. For example, turkey vultures (*Cathartes aura)* achieve faster speeds and travel more hours each day during the pre-breeding compared to the post-breeding migration because thermal uplift is stronger during the former^12^. Oriental honey buzzards (*Pernis ptilorhynchus*) traverse ecological barriers (the East China Sea) during post-breeding migration when tailwinds are available and circumvent them during pre-breeding migration when wind conditions are less favourable for sea-crossing^13^. Considering internal factors, age and experience are important factors mediating the response to weather conditions (e.g., golden eagles, *Aquila chrysaetos*^14^; ospreys, *Pandion haliaetus*^15^; black kites, *Milvus migrans*^16^; and honey buzzards, *Pernis apivorus*^17^), whereas sex typically has a small effect on travel speed of soaring migrants^18,19^.

We still know little about the relative importance of external and internal factors in shaping the migratory movements of species that can switch between flight modes, the so-called flight generalists^20^, such as bee-eaters, falcons, and harriers^21–23^. Due to their wing morphology and intermediate body size, flight generalists can use a range of flight modes in response to environmental variability^20^. Although atmospheric conditions, especially wind, significantly impact flight speeds and costs in all flying animals^21,24^, flight generalists are highly manoeuvrable and may be less constrained by suitable atmospheric conditions than obligate-soaring birds^25^. Thus, we might expect internal factors and the underlying geography to have a dominant role in shaping their migratory behaviour^20^.

Flight generalist migrants are capable of long-distance flapping, allowing them to extend their daily travel schedule into the night when thermals are weak or rare^23^. Similarly, they are capable of long sea-crossings that are generally avoided by large soaring birds (but see^26,27^). Flight generalists typically also achieve higher travel speed during nocturnal than diurnal migration, enabling them to cross ecological barriers in non-stop flights (“sprints”^28^). For example, Amur falcons (*Falco amurensis*) undertake the longest non-stop water crossing of any bird of prey studied so far, taking 3–4 days to cross the Indian Ocean, from India to East Africa (ca. 3000–4000 km) flying day and night^21^. Nevertheless, birds that invest energy in flapping flight at some point have to refuel by foraging. They may do this before or after migration, but they often do it during migration by making stopovers^4^ or by intermittent diurnal fly-forage behaviour (a combination of foraging and flying in the migratory direction^29,30^). Studies on migrants such as Eurasian hobbies (*Falco subbuteo*) and Eleonora’s falcons (*Falco eleonorae*) revealed significant seasonal variation in travel speed between regions, with fast and long flights over barriers and slower and shorter daily flights over non-barrier areas^29^. For the latter species, geography was found to have a greater influence on flight speed relative to wind and age^31,32^.

We focus on a flight generalist raptor, the lesser kestrel (*Falco naumanni*), a small-sized falcon with reverse size dimorphism (females being ca. 15% heavier than males)^33^. We investigate which are the most influential factors driving differences in travel speed, distance and duration as proxies to measure migratory behaviour at coarse (trip) and fine (daily, hourly) temporal scales. European-breeding lesser kestrels regularly perform seasonal migrations to and from sub-Saharan Africa. We describe migration patterns by investigating differences between geographies (barriers such as sea and desert, and non-barrier areas), during diurnal and nocturnal flights, accounting for season and sex. Crossing the sea and desert poses different challenges for migrants (e.g., extreme temperatures over the desert vs. few landing opportunities over the sea^34^) to which birds likely respond in different ways. Moreover, seasonal differences in travel speed are affected not only by different external conditions between seasons (e.g., wind regimes, food resources, daily cycle) while travelling over different geographies^7,35^, but also by seasonal differences in individual motivation^36^. For example, during pre-breeding migration, early arrivals can confer a reproductive advantage to adult males that compete to establish territories^6,37^.

We aim to disentangle the compounding effects of external factors (wind, geography), internal factors (sex) and season in shaping migratory behaviour. We expect tailwinds along the kestrels’ routes to explain a large part of the seasonal variation in travel speed. We further hypothesise that sex and season have a greater influence in moulding migratory behaviour compared to external factors in this flight generalist species. We predict that after accounting for wind effects, (1) the pre-breeding migration of kestrels will have a shorter duration than the post-breeding migration because of the greater selective pressure for early arrival to the breeding grounds. As such, we expect kestrels to have fewer non-travelling days, straighter routes, faster travel speed, longer daily distance, and more travel hours per day during pre- than post-breeding migration. We also predict (2) significantly higher travel speed for the smaller males than for the larger females because flapping is theoretically less costly for the former^38^, and competition for securing a high-quality territory is weaker in the latter^39^. Finally, we hypothesise that lesser kestrels will sprint over barriers (such as the Mediterranean Sea or the Sahara Desert) where there are few or no resting/drinking/feeding opportunities^23,29,34^. We thereby predict (3) that individuals will show geography-dependent differences in daily travel schedules and speeds by travelling faster, covering larger distances, and migrating at night when flying over barriers.

## Results

### Trip scale: season and sex patterns in travel duration

**Figure 1:**
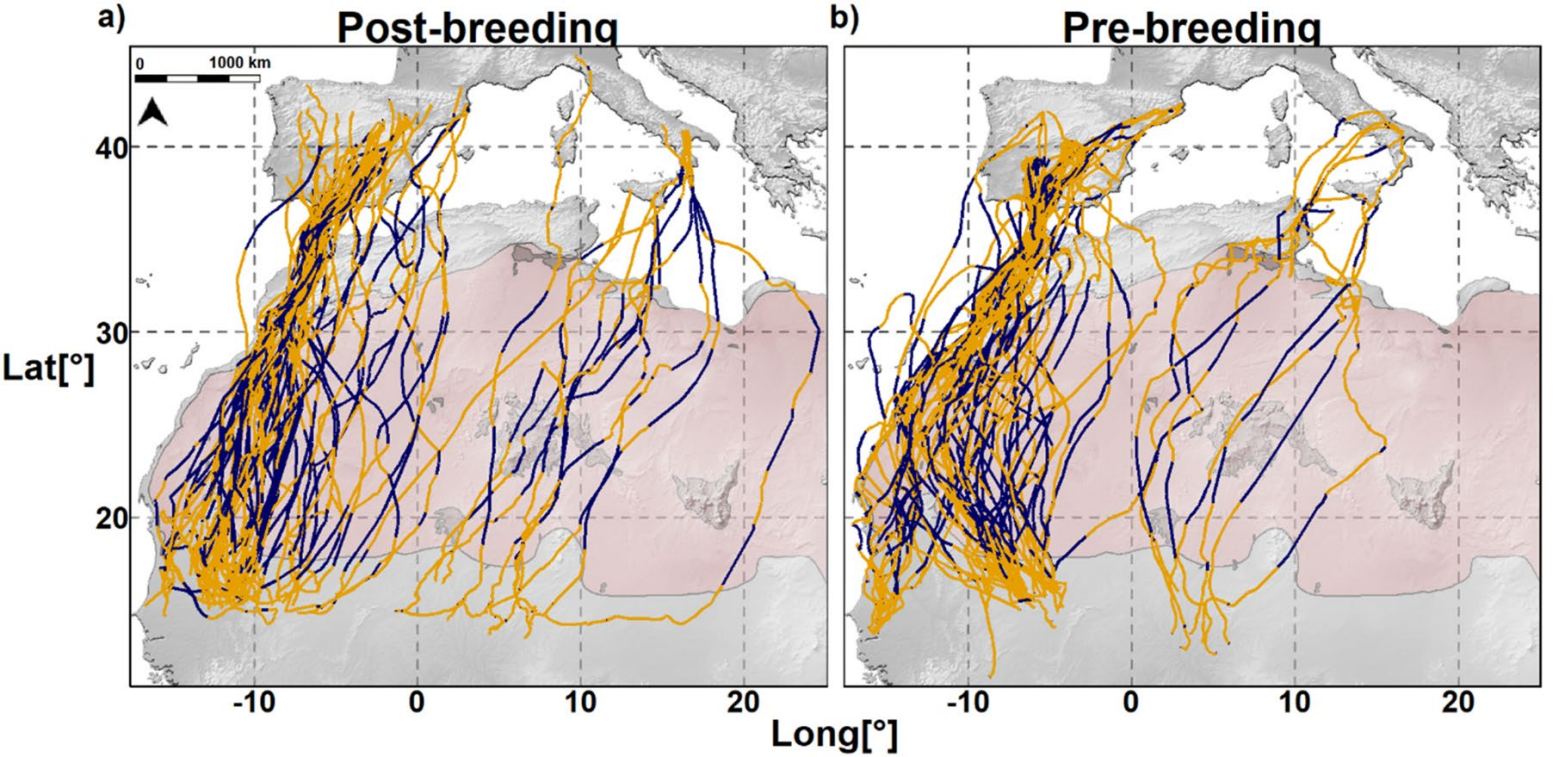
Migration routes of lesser kestrels tracked with GPS between 2014 and 2019. (**a**) Post-breeding and (**b**) pre-breeding migration. Colours indicate nocturnal migration (blue segments) and diurnal migration (orange segments) when flying over non-barriers (grey) or barriers (desert =pink, sea = white). One position per hour was plotted.

**Figure 2:**
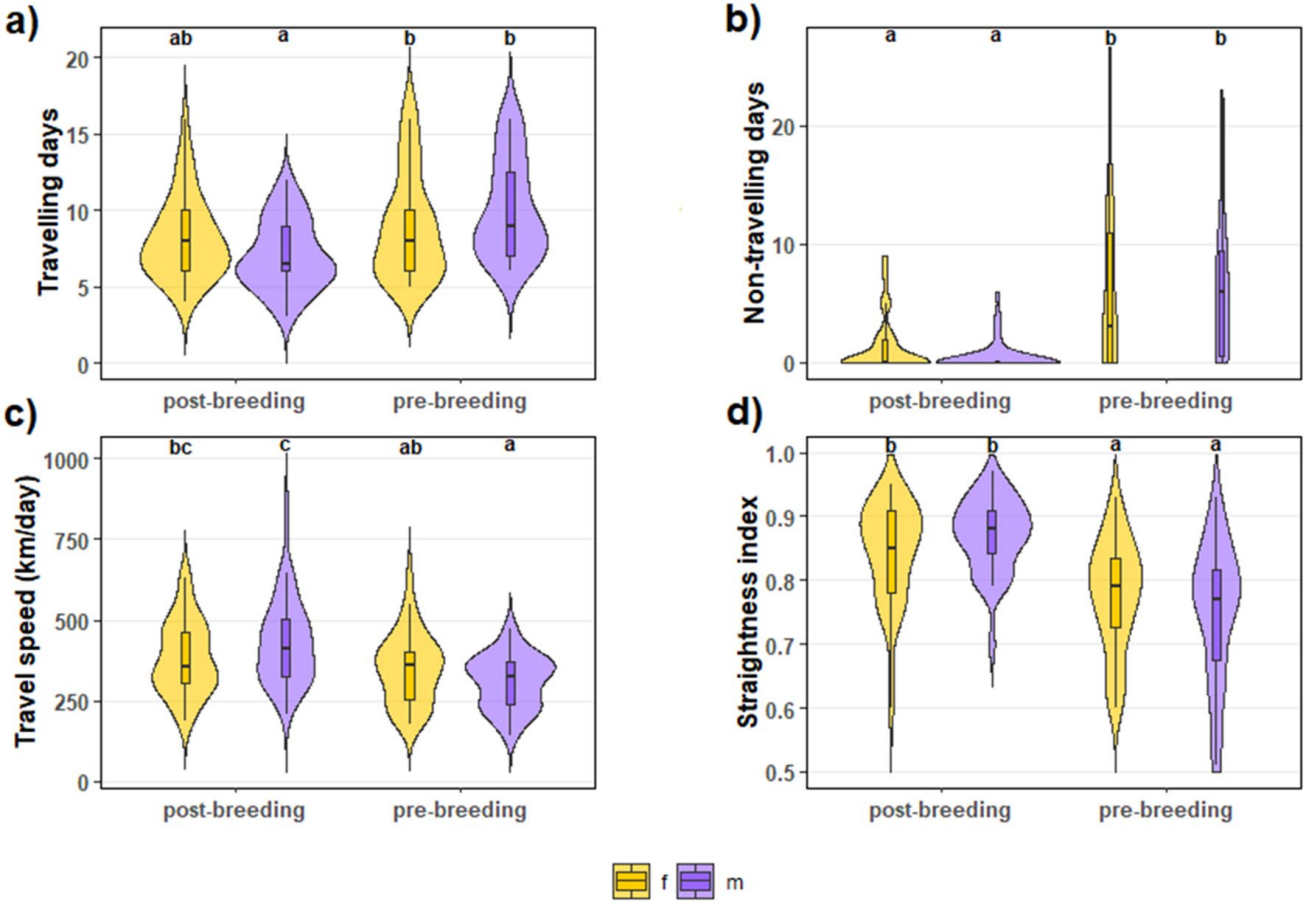
Distribution of travelling days (**a**), non-travelling days (**b**), travel speed (**c**) and straightness index (**d**) of lesser kestrels accounting for season and sex (females in yellow, males in purple). The letters above represent significant differences by Tukey HSD *post-hoc* tests at the 0.05 significance level. Groups sharing the same letter are not significantly different.

We obtained GPS data for 141 (75 post-breeding and 66 pre-breeding) complete migratory trips from 70 adults (Fig. 1). Contrary to theoretical predictions, but consistent with previous findings^40^, our data showed that birds progressed significantly faster during the post-breeding than during the pre-breeding migration (405 ± 14.33 km/day vs. 331.03 ± 12.21 km/day, respectively, excluding non-travelling days, *p* ≤ 0.05—see Supplementary Table S1 and Fig. 2). Migration was significantly shorter during the post-breeding than during the pre-breeding migration (trip duration in days: 8.62 ± 0.44 vs. 15.62 ± 1.04, *p* ≤ 0.05). They showed significantly fewer non-travelling days and followed straighter paths during the post-breeding than during the pre-breeding migration (non-travelling days: 1.00 ± 0.23 vs. 6.00 ± 0.78; straightness index: 0.86 ± 0.01 vs. 0.76 ± 0.01, respectively, *p* ≤ 0.05). Seasonal migratory behaviour was similar between sexes.

### Daily scale: geographical patterns in migratory behaviour

In agreement with predictions on geography-dependent behaviour, we observed substantial differences in migratory behaviour between barriers and non-barriers. (Supplementary Table S2a). Lesser kestrels travelled faster, covered longer straight-line distances and travelled more hours per day over barriers. Travel speed and straight-line distance were not significantly different over the sea and the desert (*p* ≥ 0.05). However, daily travel duration was significantly higher over the sea (20.00 ± 1.63 h) compared to the desert (12.72 ± 0.45 h) (sample size = 783 bird-migration days from sunrise to sunrise of the next day) (Supplementary Table S2b).

### Daily scale: season, sex and external factors

Contrary to our hypothesis, season and sex had a limited role in modulating daily migratory behaviour. Rather, external factors explained the largest amount of seasonal and daily variation in migratory behaviour. Neither sex nor the interaction effect between season and sex were significant in any of the models (Table 1 and Supplementary Table S3).

**Table 1:** Estimates for fixed effects on daily mean travel speed, travel straight-line distance and travel duration as estimated by the most parsimonious model when flying over barrier (sample size = 183 travel days) or non-barrier (sample size = 600 travel days) areas.

Response	Model	Predictor	Estimate	SE	t/z
Speed (km/h)	Over barriers	Intercept	− 0.07	0.08	− 0.91
		Nocturnal trav. fraction	0.57	0.06	9.62***
		Tailwind	0.33	0.05	7.21***
	Over non-barriers	Intercept	− 0.07	0.06	− 1.25
		Nocturnal trav. fraction	0.40	0.03	11.76***
		Tailwind	0.33	0.04	8.82***
		Crosswind	0.25	0.05	5.31***
		Season (Pre-breeding)	− 0.14	0.06	− 2.19*
Straight-line distance (km)	Over barriers	Intercept	− 0.02	0.05	− 0.48
		Nocturnal trav. hours	0.68	0.04	18.25***
		Tailwind	0.27	0.03	9.33***
		Diurnal trav. hours	0.24	0.04	5.65***
	Over non-barriers	Intercept	− 0.03	0.02	− 1.21
		Nocturnal trav. hours	0.68	0.02	41.76***
		Diurnal trav. hours	0.30	0.01	21.03***
		Tailwind	0.21	0.02	12.26***
		Season (Pre-breeding)	0.06	0.03	2.29*
Travel duration (h)	Over barriers	Intercept	2.62	0.08	34.77***
		Season (Pre-breeding)	− 0.27	0.06	− 4.29***
		Barrier type (Sea)	0.23	0.10	2.33*
		BLH	− 0.20	0.03	− 6.18***
		Tailwind	0.12	0.02	6.50***
		Crosswind	0.10	0.03	3.38***
	Over non-barriers	Intercept	2.15	0.03	66.54***
		Season (Pre-breeding)	− 0.30	0.03	− 8.59***
		Crosswind	0.29	0.02	15.64***
		Tailwind	0.11	0.02	6.28***
		BLH	− 0.09	0.02	− 5.18***

The models for travel duration included the factor barrier type with two levels: sea and desert. Boundary layer height (BLH) serves as a proxy for the availability and strength of thermal uplifts. Model estimates in units of standard deviation (SD) (organised from higher to lower relative importance), standard error (± SE), and the t-value and z-value (the ratio between the estimate and its SE) are given. All models included individual identity (ID) as a random effect. (*p ≤ 0.05; **p ≤ 0.01; ***p ≤ 0.001).

The most parsimonious model for mean travel speed when flying over barriers retained nocturnal travel fraction and tailwind, with positive effects and this was consistent in both seasons (see Supplementary Fig. S1a, b). Over non-barriers, the most influential variables determining speeds were nocturnal travel fraction and winds, with positive effects.

For straight-line distance, the most parsimonious model when flying over barriers and non-barriers retained nocturnal and diurnal travelling hours and tailwind, with a strong positive effect of nocturnal travelling. We found a positive effect of tailwind, with birds flying farther with stronger mean daily tailwind, and this effect was weaker over barriers during the pre-breeding migration (see Supplementary Fig. S1c, d).

For travel duration, the most parsimonious model when flying over barriers and non-barriers retained wind, boundary layer height (hereafter BLH) and season. Travel duration of lesser kestrels was negatively associated with mean BLH and positively associated with absolute crosswind and tailwind strength. Barrier type was retained, indicating more extended travel duration over the sea relative to the desert. We also found substantial seasonal effects with birds travelling fewer hours per day during pre-breeding relative to the post-breeding migration.

### Hourly scale: season, geography and travel schedules

**Figure 3:**
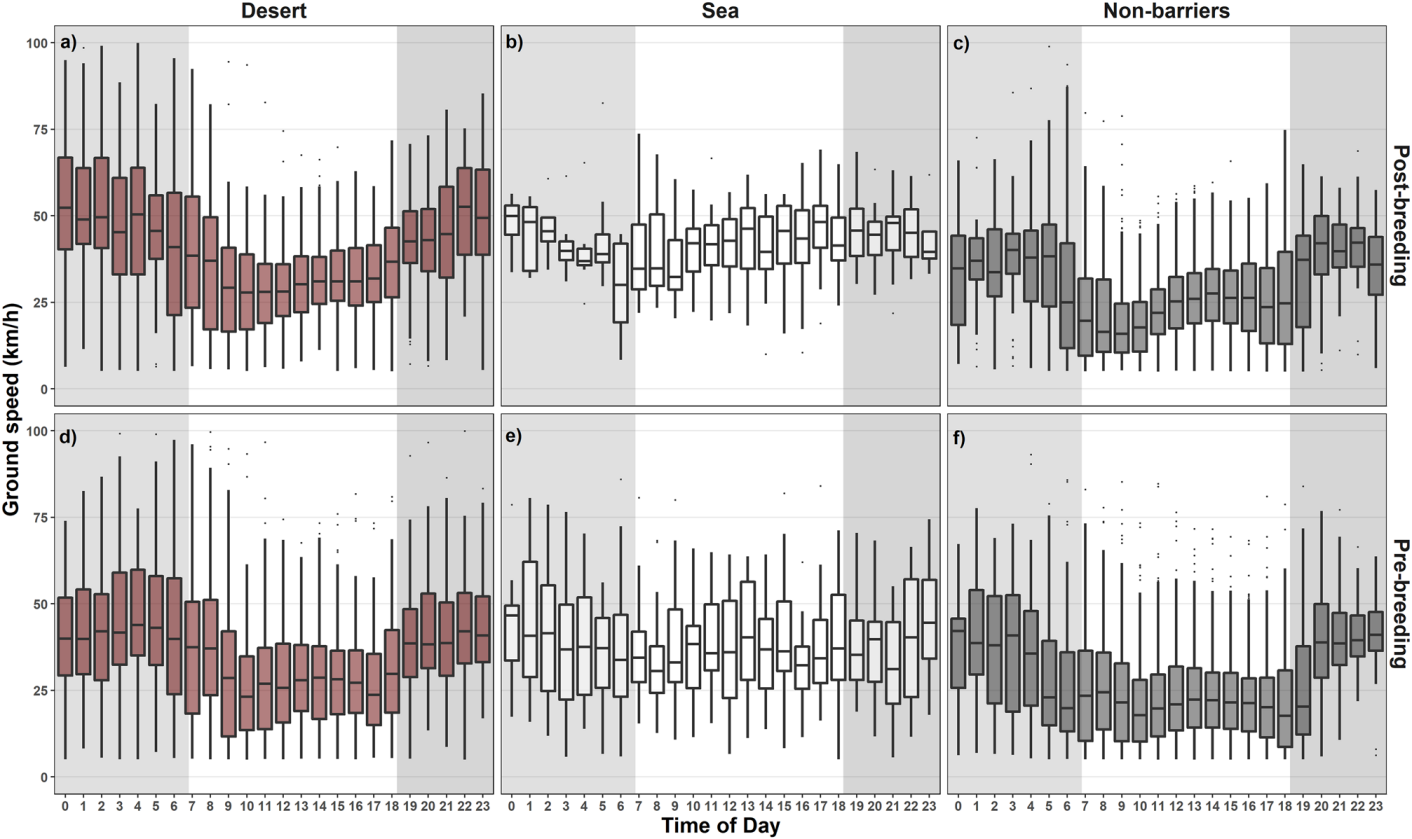
Ground speed during the post-breeding migration over barriers (**a**) desert and (**b**) sea, and (**c**) non-barriers and during the pre-breeding migration over barriers (**d**) desert, (**e**) sea, and (**f**) non-barriers. Only travel segments for each hour of the day are included. The grey areas in the background indicate nocturnal hours, and the white area indicates diurnal hours. Points represent outliers. Speed patterns are more similar when flying over the desert and non-barriers than over the sea, although over the desert, speeds are higher during the post-breeding migration, likely due to supportive winds (see Fig. 4). Over the sea kestrels achieve more constant travel speeds between ~ 25 and 50 km/h with no differences between diurnal and nocturnal flights. During diurnal travel over non-barriers travel speed typically falls below 25 km/h.

Our analyses at the hourly scale matched the behavioural patterns we observed at the daily scale. The distribution of travelling and non-travelling segments was significantly different between seasons (post-breeding vs. pre-breeding: χ^2^ = 840.63; *DF* = 1; *p* ≤ 0.05) and between geographies (barrier vs. non-barrier: χ^2^ = 658.41; *DF* = 1; *p* ≤ 0.05—see Supplementary Fig. S2), but it was not significantly different between sexes (females vs. males: χ^2^ = 2.79; *DF* = 1; *p* = 0.09) (Supplementary Table S4). Lesser kestrels attained faster travel speed during nocturnal vs. diurnal travel (Supplementary Table S4). A *post-hoc* multiple comparisons test showed the lowest speeds took place over non-barrier areas during the day (24.00 ± 0.43 km/h, *p* ≤ 0.05) and the highest speeds over the desert and sea at night (45.20 ± 0.56 km/h vs. 42.1 km/h ± 0.98 respectively, *p* ≤ 0.05) (Fig. 3 and Table 2). Over the sea, the difference in speed between diurnal and nocturnal flights was not significant (39.70 ± 0.77 vs. 42.10 ± 0.98, respectively, *p* ≥ 0.05).

**Table 2:** Summary table showing the sample size (N) and the mean (± SE) hourly speed of lesser kestrels when flying over the desert, sea and non-barriers during diurnal and nocturnal flights.

Pairwise comparison	N	Hourly speed (km/h)
Diurnal travel over non-barriers	6373	24.0 (0.43)^a^
Diurnal travel over desert	2440	31.9 (0.50)^b^
Nocturnal travel over non-barriers	1094	35.0 (0.60)^c^
Diurnal travel over sea	526	39.7 (0.77)^d^
Nocturnal travel over sea	287	42.1 (0.98)^d^
Nocturnal travel over desert	1532	45.2 (0.55)^e^

Multiple comparisons of means were performed using Tukey’s post hoc tests at the 0.05 significance level. Means sharing the same group letter are not significantly different.

### Hourly scale: season, sex and external factors

**Figure 4:**
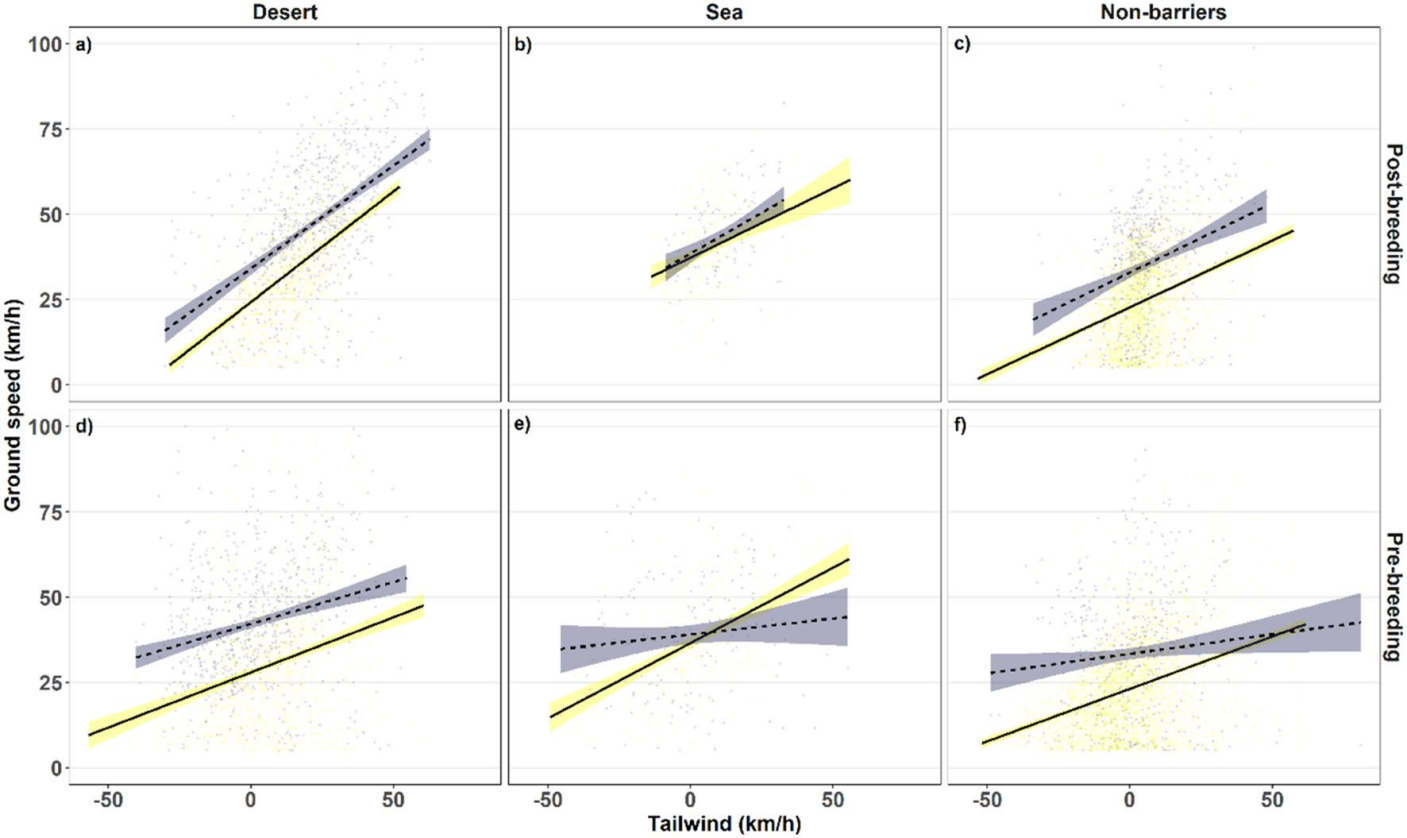
Ground speed in relation to tailwind along the kestrel’s routes accounting for season and geography. We show the linear relationship between hourly speed and tailwind (including only travel segments) and the effects during the post-breeding migration over barriers (**a**) desert and (**b**) sea, and, (**c**) non-barriers and during the post-breeding migration over (**d**) desert, (**e**) sea and (**f**) non-barriers, accounting for diurnal (solid yellow line) and nocturnal migration (blue dashed line).

The variable that had the highest positive predictive importance on ground speed was tailwind strength (Fig. 4). Absolute crosswind had a negative effect on kestrel ground speed (Table 3 and Supplementary Table S5). During diurnal flights over non-barriers, season had a small and marginally significant impact on ground speed. The interaction between season and sex was significant, indicating that the speed difference between males and females during diurnal flights over non-barriers was significantly smaller during the pre-breeding relative to the post-breeding migration.

**Table 3:** Estimates for fixed effects at the hourly scale as estimated by the most parsimonious model during diurnal migration over barriers (sample size = 2,966), nocturnal migration over barriers (sample size = 1,819), diurnal migration over non-barriers (sample size = 6,373) and nocturnal migration over non-barriers (sample size = 1094).

Model	Predictor	Estimate	SE	t	*p*
Diurnal over barriers	Intercept	1.13	0.05	24.45	0.00
	Crosswind	− 0.11	0.03	− 4.22	0.00
	BLH	− 0.10	0.01	− 7.29	0.00
	Geography (Sea)	0.41	0.04	9.23	0.00
	Tailwind	0.43	0.02	25.04	0.00
Nocturnal over barriers	Intercept	1.64	0.06	25.89	0.00
	Crosswind	− 0.13	0.04	− 3.58	0.00
	Tailwind	0.36	0.02	15.69	0.00
Diurnal over non-barriers	Intercept	0.63	0.03	20.97	0.00
	Crosswind	− 0.08	0.02	− 5.27	0.00
	BLH	0.02	0.01	2.40	0.02
	Season (Spring)	0.14	0.03	5.61	0.05
	Sex (Male)	0.07	0.04	1.67	0.10
	Tailwind	0.32	0.01	29.03	0.00
	Season:sex	− 0.18	0.04	− 4.93	0.00
Nocturnal over non-barriers	Intercept	1.27	0.06	22.68	0.00
	Crosswind	− 0.13	0.06	− 2.24	0.03
	Tailwind	0.26	0.04	6.82	0.00

Boundary layer height (BLH) serves as a proxy for the availability and strength of thermal uplifts; thus, we only included BLH in diurnal models. Model estimates in units of standard deviation (SD) (organised from higher to lower relative importance), standard errors (± SE) and the t-value (the ratio between the estimate and its SE) are given. All models included individual identity (ID) as a random effect. (*p ≤ 0.05; **p ≤ 0.01; ***p ≤ 0.001).

## Discussion

Contrary to our expectations for a flight generalist, our work suggests that migratory behaviour was only marginally influenced by sex and season. External drivers, in particular tailwinds experienced *en route*, were the main determinant of seasonal variation in travel speed, whereas geography moulded differences in daily distances by shaping daily travel schedules, with a propensity for sprinting across barriers.

In accordance with previous tracking studies on the lesser kestrel, and contrary to our first prediction, individuals completed their migration faster during the post-breeding than during the pre-breeding migration^40^. Wind explained much of this seasonal variation, i.e., birds experienced more intense tailwinds along their realised travel direction during the post-breeding compared to the pre-breeding migration. By extending travel during the night, lesser kestrels could cover up to 1000 km per day in supportive autumn winds while only 500 km through opposing spring winds. Previous work on flight generalist birds in the African-Eurasian flyway pointed to the significance of tailwinds in determining travel speed and duration, whereby prevailing winds generally opposed northward migration during the pre-breeding migration, likely causing less straight routes compared to the post-breeding^23,41,42^. We also found that crosswind strength relative to the kestrels travel direction and boundary layer height were comparatively less influential than tailwind strength on daily and hourly speeds and daily distance. Such results were expected for a flight generalist, which is not so dependent on thermals, that can alternate between flapping and soaring-gliding flight to efficiently overcome crosswinds, in contrast to larger birds that inevitably drift from their intended direction with every thermal ascent^8^. Although orientation behaviour (i.e., heading in relation to wind direction) is still to be investigated, kestrels seem rather prone to drifting in strong winds, especially above the desert^41^.

During diurnal migration over non-barriers, kestrels appear to travel slightly faster during the pre- than post-breeding migration. We envisage two mechanisms: (1) seasonality in prey availability may favour different foraging strategies at different times of the year, and (2) lesser kestrels may accelerate flight when approaching the breeding grounds if there is an urgency to arrive early to secure breeding sites^37^. Uncovering such time-selecting behaviour during the pre-breeding migration requires further study and a deeper understanding of the lesser kestrel’s settlement phase (i.e. the time between territory establishment and the onset of the breeding period^1^). Contrary to our second prediction of higher speeds for the smaller males, we did not find any sex differences in seasonal and daily migratory behaviour. Our hourly models did capture a marginal effect of the interaction between season and sex. We found that during the pre-breeding migration, males flew slower than females during diurnal migration over non-barriers relative to the post-breeding migration. However, it is important to consider that lesser kestrels reach their breeding grounds on average two months before the onset of breeding^43^. Such a long establishment phase may well offset the need for early arrivals in pre-breeding migration and favour individuals that arrive in good condition to secure territories and prepare for reproduction^44^. In that case, one would indeed expect males and females to respond similarly to weather conditions and resource availability, as did they in our study.

Our predictive variables in the hourly scale models explained relatively little variation than those at the daily scale. There are several potential limitations to the interpretation of our results. Firstly, it is likely that other external factors that we did not measure directly in this study, such as seasonal peaks in food abundance, explain spatio-temporal variation in migratory behaviour^7,11^. It should be noted that the only pure internal factor we considered was sex, and it is therefore likely that we underestimated the effects of other biometric characteristics such as body mass, wing morphology, and other internal factors such as age and breeding status, that were not available in our data set. Secondly, wind speeds are estimated by models at a coarser temporal (6 h) and spatial resolution (0.75°) than kestrel tracking data, and kestrel flight altitude varies around the 925 mb pressure level more within than between days. It is therefore likely that our weather variables are less suited to explain variation at such a fine temporal scale.

In agreement with our third prediction, lesser kestrels exhibited a propensity for sprinting when crossing barriers like the Sahara Desert or the Mediterranean Sea by travelling through the night as well as the day. When crossing barriers, birds thus showed a clear time-minimising behaviour in both seasons. We found higher mean travel speed over the sea and desert during nocturnal flights, almost twofold the travel speed over non-barriers during diurnal flights. During diurnal migration over non-barriers, travel speed typically falls below 25 km/h. We suggest that this can be due to differences in foraging opportunities and birds switching between flight-modes, as suggested for other flight generalists (gulls^22^, falcons^29^ and harriers^23^). Thermal-soaring flight is thereby expected to dominate during diurnal migration^29^ and flapping flight during nocturnal migration, although kestrels may also resort to flapping flight during the day to reduce the time needed to cross inhospitable barriers^31, 45^. Over the sea, no differences were found in travel speed between diurnal and nocturnal flights, with mean speeds between 42 and 45 km/h, which we believe is due to a consistent use of flapping flight over water. This pattern suggests that seas are a major ecological barrier not only for soaring birds^21^ but also for flight generalists, even though we cannot exclude the possibility that kestrels exploit weak sea thermals^26,27^.

## Conclusion

We conclude that lesser kestrels exhibited great behavioural plasticity in migration, sprinting through the night across barriers, and possibly engaging in fly-forage behaviour elsewhere. In all cases, however, tailwind assistance significantly increases the hourly and daily travel speed of migration, and this accounts for the faster post- than pre-breeding migration. We suggest a long establishment phase likely buffers against an internal motivation for faster pre-breeding migration in lesser kestrel males. Our study generally emphasises the importance of accounting for external factors when interpreting complex spatiotemporal movement patterns and that season and sex play a limited role in modulating migratory behaviour even in flight generalist migrants.

## Methods

### Ethical statement

All experimental protocols were approved by Estación Biológica de Doñana Ethical Committee, Consejo Superior de Investigaciones Científicas Ethical Committee, and Consejería de Agricultura, Ganadería, Pesca y Desarrollo Sostenible de la Junta de Andalucía and carried out in accordance with relevant regulations approved by the Spanish Law on Animal Experimentation (RD53/2013 from 1st February https://www.boe.es/eli/es/rd/2013/02/01/53). In Italy, procedures were approved by the regional authorities (Regione Sicilia n. 1616/2014 and Regione Puglia n. 475/20169) following the guidelines approved by the Law 157/1992 [Art.4(1) and Art 7(5)], which regulates research on wild bird species conducted mainly by the Italian Institute for Environmental Protection and Research (ISPRA). Capture and device deployment were carried out by experienced ornithologists only in accordance with approved guidelines aimed at ensuring animal welfare throughout the operations^46^. Handling time was kept to a minimum to reduce the potential for stress. No individual was injured during the capturing/handling procedure. When applicable, the design and reporting of the study were in accordance with ARRIVE Essential 10 international guidelines^47^.

### Study species and data collection

The lesser kestrel is a small insectivorous raptor, breeding in colonies across southern Europe, northern Africa to China, and with non-breeding areas in Africa, especially south of the Sahel to South Africa. However, some Mediterranean populations also contain resident individuals. From 2014 to 2019, we trapped 211 adult lesser kestrels (101 females and 110 males) at 33 breeding colonies in Spain and Italy. We fitted them with different solar GPS-UHF biologgers (Pica, Ecotone, Gdynia, Poland; Microsensory LS, Córdoba, Spain; and NanoFix GEO+RF, Pathtrack Ltd., Leeds, UK., weighing 4–5 g) attached as backpacks with a Teflon harness. Loggers plus harness did not exceed 4% of the average lesser kestrel’s weight, which is within the accepted standards for animal welfare in research^48^. Loggers were programmed with different schedules (i.e. device’s duty cycle varied from 8 to 24 h) and recorded GPS-locations day and night (65% of the tags had 24 h duty cycle). Over the whole migration, tracks were sampled with GPS fixes every 30 min to 1 h, depending on solar battery recharge and satellite geometry (≥ 4 satellites must be detected for a reliable fix). Data were stored on-board the device and downloaded the following year from successfully migrating individuals that returned to the breeding area via UHF base stations placed at the vicinity of the colony.

### Tracking data set

We included 70 adults in our analyses (40 females and 30 males) who completed their migration from Spain (n = 58) and Italy (n = 12) to Africa and back, either along the East Atlantic or the Central Mediterranean migration flyways. These birds provided 75 post-breeding and 66 pre-breeding migration trips. Of these kestrels (16/70) 23% had two and three repeated migration cycles. Of the birds that did not yield any migration track, 40 had confirmed technical failure of the tag (i.e. 23 tagged birds were seen in the colony but did not send data and 17 tags stopped providing GPS coordinates soon after deployment). Seven dispersed from the core study area, 4 were reported dead, and 2 with partial migration strategies were excluded from the analysis because their behaviour differed substantially. In the rest of the cases, we do not know the fate of the birds, but it is likely that they either died, dispersed, tags failed or were missed. One bird was seen again in 2021 after being missed during 2019 and 2020. As a consequence of all these factors, we are unable to estimate the actual impact of GPS tags on the return rate in this specific study but we cannot discount the inevitable impact of tagging.

### Identifying migratory trips

The onset and end of migration were identified based on marked shifts in daily distance histograms^8^. We calculated the distance between the current position to the previous one using the deg.dist function in the R package ‘fossil’^49^. For each migratory trip, we searched for a group of first and last three consecutive days with an average daily distance of at least 150 km, preceded (if onset) or followed (if end) by a stationary phase of five consecutive days with daily mean travel distance < 70 km^19^. We assigned as the migration start day the first day of the first three-day period and as the migration end day the last day of the last three-day period. We confirmed those dates visually using QGIS^50^. We excluded tracks in which we could not precisely determine the onset or the end of migration due to the lack of GPS fixes (four cases during the post-breeding and thirteen cases during the pre-breeding migration).

### Estimating travel metrics and their scales

At the trip scale, we defined a migratory trip as the set of data between the first position on migration start day and the last position on migration end day. We computed: (a) the migration duration as the period between the migration start and end dates; (b) the trip straight-line distance (i.e., the shortest orthodromic path) as the distance between the first position on migration start day and the last position on migration end day; (c) the cumulative distance as the sum of the successive daily travel distances between the start and end of migration dates; (d) trip straightness index as the ratio between the trip straight-line distance and the cumulative distance, ranging between 0 (corresponding to tortuous routes) and 1 (corresponding to straight-line routes); (e) the number of non-travelling days by summing days with a daily distance < 50 km (see below); (f) travel speed was defined as the ratio between the straight-line distance and the migration duration in days (excluding non-travelling days). Means are given with standard errors throughout the paper.

At the daily scale, we defined each migration day of a kestrel from sunrise to sunrise of the next day (in our dataset lesser kestrels were frequently travelling during the night), thus capturing a complete day-night cycle. Partially due to low battery power (e.g., reduced amount of solar energy that reached the telemetry unit solar panel) and different working schedules (i.e., within a range of 8–24 h, as outlined above), some data gaps within migratory travel days were detected. We selected only those days with a minimum of 75% of daily coverage for this analysis. This was done to avoid bias in the calculation of daily metrics due to significant data gaps. The number of fixes per day was 22.72 ± 2.00. We computed the following travel metrics: (a) daily straight-line distance between the first and the last position of each unit day; (b) the daily travel duration, which is the cumulative sum of hourly travel segments (excluding foraging and resting events, see below); and (c) the daily mean travel speed as the daily straight-line distance divided by the travel duration. Flying for more hours per day or night determines a lot of the variation in daily distance^45^. Since we aimed at quantifying what factors explain migratory behaviour during travel events, we calculated metrics during travel hours only and computed daily mean travel speed accounting for the effect of travel duration. Travelling days were defined as those in which a kestrel’s displacement in the direction of migration was at least 50 km^41,51^. Non-travelling days defined as complete days (sunrise to sunrise) in which less than 50 km of travel in the direction of migration was observed, were excluded from further analysis.

At the hourly scale, all data were resampled to a 1-h interval, allowing deviations up to 20 min to maximise the number of observations. By resampling, we also avoided bias in our calculations of migratory parameters due to the variability in sampling frequencies^8,52^. After resampling, we analysed 31,153 hourly segments, from which 12,252 were annotated as travel segments. We calculated the travel distance and ground speed from each resampled location to the previous. We classified as travel segments those in which speed was ≥ 5 km/h^29,53^.

### Annotating environmental variables

At the hourly scale, to examine possible changes in migratory behaviour of birds over different geographies, we first identified when GPS fixes were located over ecological barriers, specifically over the Mediterranean Sea or the Sahara Desert and over non-barriers (see Supplementary Methods for details) using the Global Biomes map^54^. We used the ‘join-attribute-by-location’ tool in QGIS^50^ to join all the tracks to the corresponding position within the Global Biomes map. To identify possible changes in the behaviour of kestrels in relation to the time of day (i.e., day and night), we used the *sunrise.set* function in R package ‘StreamMetabolism’^55^. We classified as diurnal all locations detected between sunrise and sunset, with the rest being nocturnal. To account for the influence of atmospheric conditions on migratory behaviour, we annotated each GPS fix with environmental data, namely boundary layer height (BLH) and wind using the Env-Data annotation service of Movebank^56^ (see Supplementary Methods for details). We identified daily travel schedules in relation to the hour of the day. For every migration day, the number of hourly segments was annotated according to two behaviours: travelling or non-travelling^34^.

At the daily scale, to examine how geography influences migratory behaviour, we classified migration days as desert, sea and non-barrier days based on the proportion of time kestrels spent over the same geography (≥ 60% of day). We computed the amount of diurnal and nocturnal travelling time by summing diurnal and nocturnal travel segments. As we expected distance to increase linearly with travel duration, we used those segments directly as control variables to account for differences in travel duration in the weather models. We also calculated the nocturnal travel fraction (nocturnal travelling hours/total travelling hours) and included it in the daily speed models. We calculated mean daily tailwind, absolute crosswind, and mean daily BLH by averaging across the day, using only travel segments.

### Statistical analysis

#### Trip scale

For our first and second prediction, we tested for differences in migratory behaviour between seasons and sexes using univariate statistics. After testing for normality, we used the parametric t-test for speed and non-parametric Wilcoxon rank-sum test for mean trip duration (days), mean number of travelling days, mean number of non-travelling days, and straightness index. Analyses were conducted using the ‘stats’^57^ package in R. For pairwise comparisons we used Tukey’s HSD (honestly significant differences) tests, considering an effect to be significant if *p* ≤ 0.05, conducted with the ‘emmeans’^58^ package.

#### Daily scale

For our third prediction, we identified whether there was a significant difference in mean daily migratory behaviour (i.e., travel speed, straight-line distance, and duration) among geographies (three level factor: sea, desert and non-barrier), using Generalised Linear Mixed Models (GLMMs) with bird identity as a random effect. After visual inspection of residual plots, we fitted models with Gaussian error distribution for travel speed, daily straight-line distance, and Poisson error and log link function for daily travel duration, which is appropriate for count data^59^. We conducted a Tukey’s honest significance test, using the package ‘multcomp’^60^.

To disentangle the most influential factors driving differences in migratory behaviour, we modelled the relationship between daily metrics when flying over barriers (pooling data for sea and desert) vs. non-barriers, when differences between sea and desert proved to be non-significant, accounting for the interaction between season and sex. We also included as predictors the weather variables (mean daily tailwinds, absolute crosswinds, and BLH) and the proportion of diurnal and nocturnal travelling hours. We first computed full models including all predictors. We used the ‘dredge’ function in the R package ‘MuMIn’^61^, which uses Akaike’s information criterion (AIC) to rank all possible subsets of reduced models from each full model. We selected the models if they had ΔAIC ≤ 2 units of the highest ranked-model and we retained the most parsimonious model (with the fewest parameters) because model averaging could not handle models with interaction effects^62,63^. We used the Satterthwaite’s method to estimate degrees of freedom and to obtain *p*-values using the ‘lmerTest’^64^ R package. The proportion of variance explained by the fixed effects (R_marginal_) and by both fixed and random effects (R_conditional_) was assessed using methods in Nakagawa and Schielzeth^65^.

Before fitting the GLMMs, all continuous predictors and response variables were transformed to z-scores, to compare the relative importance among predictors^66,67^. We checked for multicollinearity of weather variables and season and included only variables that were not highly correlated (r < 60). Multicollinearity was also tested by calculating variance inflation factors (VIF) for all our predictors using the ‘car’^68^ R package. Values of these were in all cases below 2.8. All the analyses were performed in the ‘lme4’^69^ package.

#### Hourly scale

To examine how ground speeds differed between diurnal and nocturnal travel when flying over different geographies, we used GLMMs (following the methods outlined above), using speed as a response variable and geography type and diurnal and nocturnal travel segments as fixed effects. We modelled the relationship between ground speeds when flying over barriers vs. non-barriers and during diurnal and nocturnal travel and included the interaction between season and sex and weather variables in our models, again using the approach outlined above.

To analyse the daily travel schedules, hourly travel speed were plotted in relation to the time of the day for each season and over the sea, desert, and non-barriers. In addition, we used the classification mentioned earlier to obtain the distribution of travelling and non-travelling segments across all 24 h of the day, which is a reasonable description of daily travel schedules^47^. We compared the proportion of travelling and non-travelling segments between seasons, sexes, over barriers, and non-barriers using the Pearson’s Chi-squared test using the ‘vcd’^70^ R package.

## Supplementary Information

**Supplementary Information.**

## Data Availability

GPS tracking data have been uploaded to Movebank under the study name: (SP-IT) Lesser Kestrel migration (www.movebank.org); all datasets used in this study are available upon request from the corresponding author. The data matrix is also public via digital.csic (https://digital.csic.es/).
