# Supplementary material for: Barrier crossings and winds shape daily travel schedules and speeds of a flight generalist
Source: Sci Rep. 2021 Jun 8;11:12044. doi: 10.1038/s41598-021-91378-x (PMC8187636; doi:10.1038/s41598-021-91378-x)
Supplement: Supplementary file 1 — Supplementary Information. [file 41598_2021_91378_MOESM1_ESM.pdf]

Supplementary information

Barrier crossings and winds shape daily travel schedules and speeds of a flight generalist

Lina Lopez-Ricaurte*, Wouter M. G. Vansteelant, Jesús Hernández-Pliego, Daniel García-Silveira, Ana Bermejo-Bermejo, Susana Casado, Jacopo G. Cecere, Javier de la Puente, Fernando Garcés-Toledano, Juan Martínez-Dalmau, Alfredo Ortega, Beatriz Rodríguez-Moreno, Diego Rubolini, Maurizio Sarà, Javier Bustamante*

* corresponding author:

lina@ebd.csic.es and jbustamante@ebd.csic.es

CONTENT

Supplementary Table S1. Season and sex patterns at the trip scale.

Supplementary Table S2a. Geographical patterns in migratory behaviour at the daily scale.

Supplementary Table S2b. Summary statistics for daily mean travel speed, travel straight-line distance and travel duration over different geographies (n = 783 travel days).

Supplementary Table S3. Best ranking models for season, sex and external factors at the daily scale.

Supplementary Table S4. Barriers and seasonal patterns in hourly speed of males and females kestrels.

Supplementary Table S5. Best ranking models for season, sex and external factors at the hourly scale.

Supplementary Figure S1. Linear relationships between average daily tailwind strength along the falcons realised travel direction (km/h) and lesser kestrel daily mean travel speed and straight-line distance.

Supplementary Figure S2. Travel schedules for geography and seasons.

Supplementary Methods. Annotating environmental data.

Supplementary Table S1. Season and sex patterns at the trip scale. Summary table showing sample size (N) and the mean (±SE) of the seasonal estimates for travelling days, non-travelling days, straightness index and travel speed for males and females lesser kestrels. Comparison of means performed using Tukey's *post hoc* tests at the 0.05 significance level.

	**Sex**		**Travelling days**	**Non-travelling days**	**Straightest index**	**Travel speed (km/days)**
		N	Mean (SE)	Mean (SE)	Mean (SE)	Mean (SE)
Post-breeding	Male	34	7.05[a] (0.38)	0.50[a] (0.22)	0.87[b] (0.01)	431.40[c] (22.63)
	Females	41	8.17[ab] (0.42)	1.34[a] (0.37)	0.84[b] (0.01)	383.64[bc] (17.85)
Pre-breeding	Males	31	10.06[b] (0.57)	6.54[b] (1.16)	0.75[a] (0.01)	309.54[a] (14.89)
	Females	35	8.94[b] (0.58)	5.80[b] (1.08)	0.77[a] (0.01)	350.07[ab] (18.48)

Table S2a. Geographical patterns in migratory behaviour at the daily scale. Models for mean daily travel speed, straight-line distance and duration of lesser kestrel accounting for the effect of geography (desert, sea and non-barriers) as estimated by GLMMs, including ID as a random effect (n=783 travel days, individuals=45). Also shown the estimates, standard errors (±SE), the t-value, z-value and the $R^2_{marginal,}$ which is the variation explained by fixed effects and $R^2_{conditional}$, the variation explained by the fixed and random effects. (* = p ≤ 0.05, ** = p ≤ 0.01, *** = p ≤ 0.001).

**Response**	**Predictor**	**Estimate**	**SE**	**t/z**	**R^2_{mar}**	**R^2_{con}**
Travel speed (km/h)	Intercept	28.89	1.20	24.08***	0.04	0.09
	Geography(Non-barriers)	-6.62	1.21	-5.45***		
	Geography(Sea)	3.06	4.45	0.69		
Travel straight-line distance (km)	Intercept	431.96	22.18	19.48***	0.09	0.13
	Geography(Non-barriers)	-180.89	22.89	-7.90***		
	Geography(Sea)	140.95	84.06	1.68		
Travel duration (h)	Intercept	2.54	0.04	71.36***	0.17	0.37
	Geography(Non-barriers)	-0.34	0.03	-12.43***		
	Geography(Sea)	0.45	0.08	5.62***		

Table S2b. Summary statistics for daily mean travel speed, travel straight-line distance and travel duration over different geographies (n = 783 travel days). We show the mean (±SE) for daily travel of lesser kestrels over the desert, sea and non-barriers. Groups sharing the same letter are not significantly different **(**GLMMs followed by Tukey HSD *post-hoc* test; p ≤ 0.05).

**Response**	**Speed (km/h)**	**Straight-line distance (km)**	**Travel duration (h)**
Desert	28.9 (1.21)[b]	432 (22.4)[b]	12.72 (0.45)[c]
Sea	31.9 (4.37)[b]	573 (82.4)[b]	20.00 (1.63)[b]
Non-barriers	22.3 (0.77)[a]	251 (14.0)[a]	9.09 (0.27)[a]

Supplementary Table S3. Best ranking models for season, sex and external factors at the daily scale. Selecting final GLMMs for daily mean travel speed, travel straight-line distance and travel duration as a function of the interaction between season and sex, nocturnal travel and external factors (mean daily tailwind, absolute crosswind and boundary layer height, BHL) when flying over barriers vs. non-barriers (n=783 travel days, individuals=45). All models included ID as a random effect. We show the best ranking models ($\Delta AIC \leq 2$), the models evaluating the effect of the interaction between season and sex, their AIC and the variation explained by fixed effects (R^2_{con}) and fixed and random effects (R^2_{mar}).

Response variable	Model	Fixed and random effects	df	AIC	ΔAIC	R^2_{con}	R^2_{mar}
Daily speed (km/h)	Over barriers	~ Nocturnal trav. fraction + Season + Sex + Tailwind + (1 | ID)	7	459.6	0	0.53	0.53
		~ Nocturnal trav. fraction + Season + Tailwind + (1 | ID)	6	460.6	0.90	0.52	0.53
		~ Nocturnal trav. fraction + Sex + Tailwind + (1 | ID)	6	460.8	1.11	0.52	0.52
		~ Crosswind + Nocturnal trav. fraction + Season + Sex + Tailwind + (1 | ID)	8	460.5	1.15	0.53	0.53
		~ Crosswind + Nocturnal trav. fraction + Season + Sex + Tailwind + (1 | ID)	7	460.8	1.2	0.52	0.52
		~ Crosswind + Nocturnal trav. fraction + Tailwind + (1 | ID)	6	461.2	1.5	0.51	0.53
		~ Nocturnal trav. fraction + Tailwind + (1 | ID)	5	461.4	1.5	0.51	0.52
		~ Nocturnal trav. fraction + Season * Sex + Tailwind + (1 | ID)	8	461.1	1.6	0.53	0.53
		Crosswind + Nocturnal trav. fraction + Season + Tailwind + (1 | ID)	7	461.4	1.84	0.52	0.53
		~ Season * Sex + (1 | ID)	6	579.4	119	0.03	0.13
	Over non-barriers	~ Crosswind + Nocturnal trav. fraction + Season * Sex + Tailwind + (1 | ID)	6	1276	0	0.41	0.44
		~ Crosswind + Nocturnal trav. fraction + Season + Tailwind + (1 | ID)	7	1277	1.20	0.40	0.43
		~ Crosswind + Nocturnal trav. fraction + Season + Tailwind + BHL + (1 | ID)	10	1277	1.58	0.41	0.44
		~ Season * Sex + (1 | ID)	6	1573	296	0.02	0.05
Daily straight-line distance (km)	Over barriers	~ Diurnal trav. hours + Nocturnal trav. hours + Tailwind + (1 | ID)	6	274.1	0	0.84	0.85
		~ Diurnal trav. hours + Nocturnal trav. hours +Season + Tailwind + (1 | ID)	7	247.4	0.50	0.84	0.85
		~ Season * Sex + (1 | ID)	6	583.4	309	0.05	0.29
	Over non-barriers	~ Diurnal trav. hours + Nocturnal trav. hours + Season + Sex + Tailwind + (1 | ID)	8	328.8	0	0.86	0.86
		~ Crosswind + Diurnal trav. hours + Nocturnal trav. hours + Season + Sex + Tailwind + (1 | ID)	9	329.3	0.55	0.86	0.86
		~ Diurnal trav. hours + Nocturnal trav. hours + Season + Tailwind + (1 | ID)	7	329.7	0.77	0.86	0.86
		~ Crosswind + Diurnal trav. hours + Nocturnal trav. hours + Season + Tailwind + (1 | ID)	8	330.1	1.22	0.86	0.86
		~ Diurnal trav. hours + Nocturnal trav. hours + Season * Sex + Tailwind + (1 | ID)	9	330.2	1.41	0.86	0.86
		~ BLH + Diurnal trav. hours + Nocturnal trav. hours + Season + Sex + Tailwind + (1 | ID)	9	330.3	1.55	0.86	0.86
		~ Crosswind + Diurnal trav. hours + Nocturnal trav. hours + Season * Sex + Tailwind + (1 | ID)	10	330.7	1.97	0.86	0.86
		~ Season * Sex + (1 | ID)	6	1503	1174	0.02	0.02
Daily travel duration (h)	Over barriers	~ BLH + Crosswind + Season + Tailwind + (1 | ID)	6	1252	0	0.30	0.68
		~ BLH + Crosswind + Season + Sex + Tailwind + (1 | ID)	7	1254	1.78	0.31	0.69
		~ Season * Sex + (1 | ID)	5	1365	112	0.05	0.67
	Over non-barriers	~ BLH + Crosswind + Season + Tailwind + (1 | ID)	6	3764	0	0.33	0.44
		~ BLH + Crosswind + Season + Sex + Tailwind + (1 | ID)	7	3765	1.22	0.33	0.44
		~ Season * Sex + (1 | ID)	5	4097	333	0.07	0.30

Supplementary Table S4. Barriers and seasonal patterns in hourly speed of males and females kestrels. Summary table showing the mean (±SE) hourly speed of lesser kestrels for both post- and pre-breeding migration, specifically comparing barriers vs. non-barriers and diurnal and nocturnal flight segments. Comparison of means was performed using Tukey's *post hoc* tests at the 0.05 significance level. Means sharing the same group letter are not significantly different.

	**Diurnal/ Nocturnal**	**Season**	**Sex**	**Hourly speed (km/h)**
**Barrier**	Diurnal	post-breeding	f	35.01 (0.58)^{def}
	Diurnal	pre-breeding	f	28.95 (0.64)^{c}
	Diurnal	post-breeding	m	34.24 (0.74)^{de}
	Diurnal	pre-breeding	m	28.14 (0.69)^{c}
	Nocturnal	post-breeding	f	43.60 (0.81)^{i}
	Nocturnal	pre-breeding	f	39.09 (1.03)^{gh}
	Nocturnal	post-breeding	m	43.27 (0.93)^{hi}
	Nocturnal	pre-breeding	m	38.41 (0.90)^{efg}
**Non-Barrier**	Diurnal	post-breeding	f	24.43 (0.26)^{ab}
	Diurnal	pre-breeding	f	24.84 (0.30)^{ab}
	Diurnal	post-breeding	m	26.31 (0.33)^{bc}
	Diurnal	pre-breeding	m	22.38 (0.29)^{a}
	Nocturnal	post-breeding	f	36.26 (0.82)^{defg}
	Nocturnal	pre-breeding	f	35.04 (0.87)^{def}
	Nocturnal	post-breeding	m	38.98 (0.73)^{fg}
	Nocturnal	pre-breeding	m	34.60 (0.76)^{d}

Abbreviations: f, female; m, male

Supplementary Table S5. Best ranking models for season, sex and external factors at the hourly scale. Selecting final GLMMs for hourly speed as a function of season, sex and external factors (tailwind, crosswind and BLH). All models included ID as a random effect. Only the results of the best ranking models (∆**AIC ≤ 2),** the models evaluating the effect of the interaction (season:sex), their AIC and the variation explained by fixed effects (**R^2_{con}**) and fixed and random effects (**R^2_{mar}**) are shown.

**Model**	**Fixed and random effects**	**df**	**AIC**	**∆AIC**	**R^2_{con}**	**R^2_{mar}**
**Diurnal over barriers**	~ BLH + Crosswind + Geography + Season + Tailwind + (1 | ID)	8	7062	0	0.20	0.29
	~ BLH + Crosswind + Geography + Season + Sex + Tailwind + (1 | ID)	9	7064	1.34	0.20	0.29
	~ BLH + Crosswind + Geography + Tailwind + (1 | ID)	7	7064	1.65	0.20	0.29
	~ Season * Sex + (1 | ID)	6	7742	680	0.01	0.13
**Nocturnal over barriers**	~ Crosswind + Tailwind + (1 | ID)	5	4969	0	0.13	0.23
	~ Season * Sex + (1 | ID)	6	5189	231	0.02	0.15
**Diurnal over non-barrier**	~ BLH + Crosswind + Geography + Season * Sex + Tailwind + (1 | ID)	9	12869	0	0.12	0.16
	~ Season * Sex + (1 | ID)	6	13667	797	0.01	0.06
**Nocturnal over non-barriers**	~ Crosswind + Tailwind + (1 | ID)	5	3106	0	0.04	0.10
	~ Crosswind + Sex + Tailwind + (1 | ID)	6	3108	1.7	0.04	0.10
	~ Crosswind + Season + Tailwind + (1 | ID)	6	3108	1.9	0.04	0.10
	~ Season * Sex + (1 | ID)	6	3148	42.0	0.01	0.07

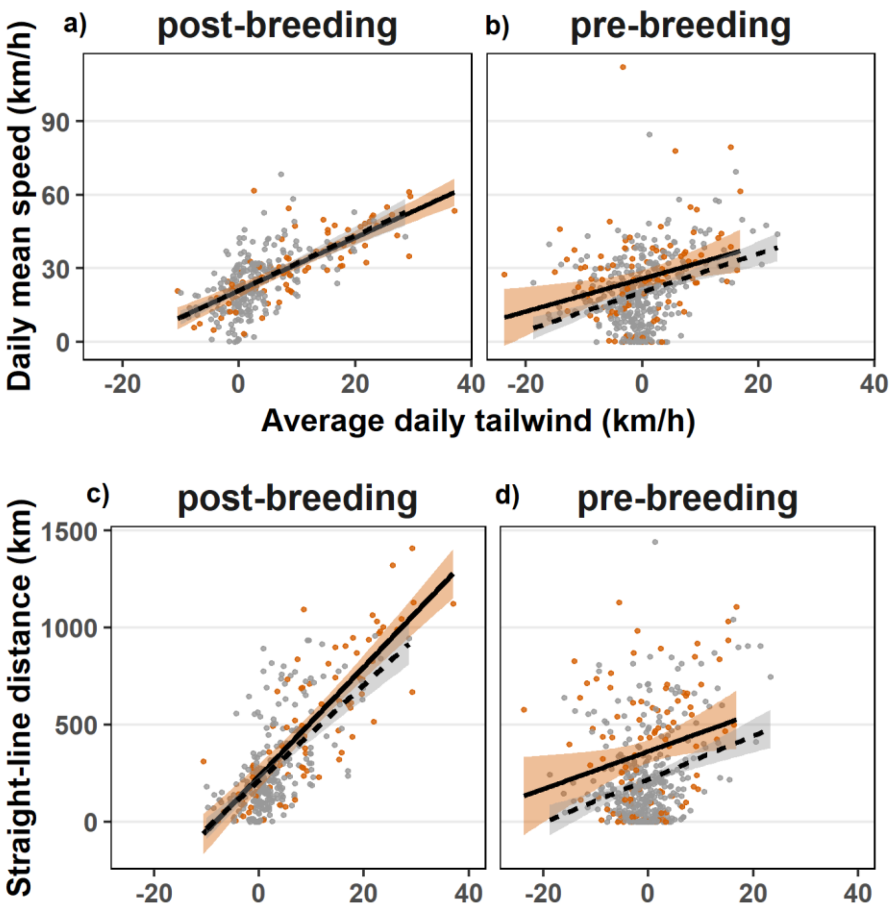

Supplementary Figure S1. Linear relationships between average daily tailwind strength along the falcons realised travel direction (km/h) and lesser kestrel daily mean travel speed and straight-line distance. (a) mean daily speed during the post-breeding migration, (b) mean daily speed during the pre-breeding migration, (c) daily straight-line distance during the post-breeding and (d) daily straight-line distance during the pre-breeding, when flying over barriers (orange and solid line) or non-barriers (grey and dashed line).

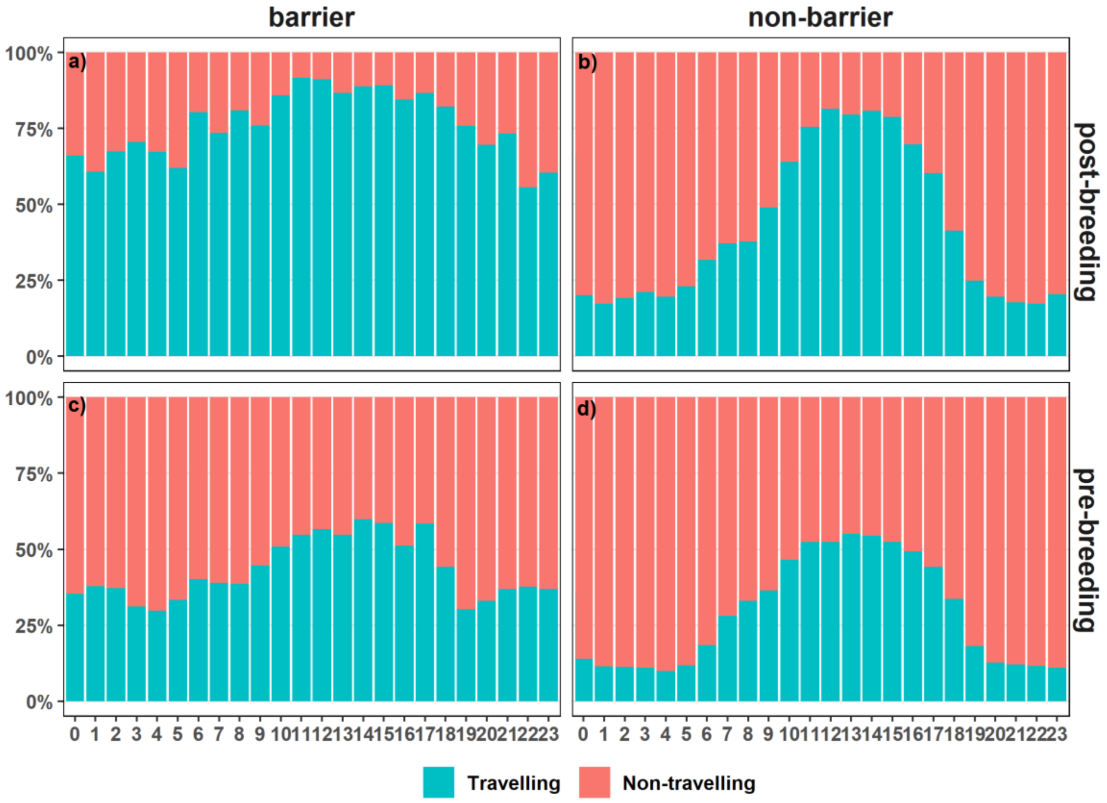

Supplementary Figure S2. Travel schedules for different geographies (barrier vs. non-barrier) and seasons. The distribution of travelling (blue) vs. non-travelling (pink) flight segments of the lesser kestrel per hour of the day during the post-breeding migration over (a) barriers and (b) non-barriers, and during the pre-breeding migration over (c) barrier and (d) non-barrier. Data over the sea and desert are pooled.

Supplementary Methods. Annotating environmental data.

Geographies

During their migratory trip, lesser kestrels pass in broad fronts over two types of barriers, the Mediterranean Sea and the Sahara Desert[1]. These are barriers for lesser kestrel migration not only due to the lack of feeding opportunity but due to lack of landing opportunities (sea) and very hostile climate (desert). Although over the sea there is also a weaker formation of thermal updrafts compared to the desert. We assigned each GPS fix to three geography categories, flying over the desert, sea and flying outside these two regions to non-barriers.

Weather effects

Each GPS fix was annotated with environmental data of wind and boundary layer height using the Env-DATA track annotation tool of MoveBank[2]. For each GPS point, we obtained: the boundary layer height (in km) (BLH), an estimate for thermal updraft formation at a spatial resolution of 0.75 degrees and temporal resolution of 3 hours; and the U (west-east) and V (north-south) wind components (km/h) at a spatial resolution of 0.75 degrees and temporal resolution of 6 hours from the ECMWF (European Center for Medium-Range Weather Forecast). To compute hourly tailwind and absolute crosswind, V- wind and U-wind components were combined in a single vector adding hourly flight direction in degrees to the north and wind strength[3]. We determined tailwind strength and absolute crosswind strength relative to the realised hourly travel direction of migration. We used weather data from the 925 hPa pressure level, corresponding to a mean flight altitude between 445 and 1,145 m a.s.l., which has been used extensively throughout migratory raptor studies[4,5]. We selected the bilinear interpolation method for all wind variables.

References

1. Sarà, M. *et al.* Broadfront migration leads to strong migratory connectivity in the lesser kestrel (*Falco naumanni*). *J. Biogeogr.* **46,** 2663-2677 (2019).

2. Dodge, S. *et al.* The environmental-data automated track annotation (Env-DATA) system: linking animal tracks with environmental data. *Mov. Ecol.* **1,** (2013).
3. Vansteelant, W.M.G. et al. Regional and seasonal flight speeds of soaring migrants and the role of weather conditions at hourly and daily scales. *J. Avian Biol.* **46,** 25–39 (2015).

4. Schmaljohann, H., Fox, J. W. & Bairlein, F. Phenotypic response to environmental cues, orientation and migration costs in songbirds flying halfway around the world. *Anim. Behav.* **84,** 623–640 (2012).

5. Limiñana, R., Romero, M., Mellone, U. & Urios, V. Is there a different response to winds during migration between soaring and flapping raptors? An example with the Montagu's harrier and the lesser kestrel. *Behav. Ecol. Sociobiol.* **67,** 823–835 (2013).
